# Poldip2 Knockout Results in Perinatal Lethality, Reduced Cellular Growth and Increased Autophagy of Mouse Embryonic Fibroblasts

**DOI:** 10.1371/journal.pone.0096657

**Published:** 2014-05-05

**Authors:** David I. Brown, Bernard Lassègue, Minyoung Lee, Rostam Zafari, James S. Long, Harold I. Saavedra, Kathy K. Griendling

**Affiliations:** 1 Department of Medicine, Division of Cardiology, Emory University School of Medicine, Atlanta, Georgia, United States of America; 2 Department of Radiation Oncology, Emory University School of Medicine, Atlanta, Georgia, United States of America; McGill University, Canada

## Abstract

Polymerase-δ interacting protein 2 (Poldip2) is an understudied protein, originally described as a binding partner of polymerase delta and proliferating cell nuclear antigen (PCNA). Numerous roles for Poldip2 have been proposed, including mitochondrial elongation, DNA replication/repair and ROS production via Nox4. In this study, we have identified a novel role for Poldip2 in regulating the cell cycle. We used a *Poldip2* gene-trap mouse and found that homozygous animals die around the time of birth. *Poldip2−/−* embryos are significantly smaller than wild type or heterozygous embryos. We found that *Poldip2−/−* mouse embryonic fibroblasts (MEFs) exhibit reduced growth as measured by population doubling and growth curves. This effect is not due to apoptosis or senescence; however, *Poldip2−/−* MEFs have higher levels of the autophagy marker LC3b. Measurement of DNA content by flow cytometry revealed an increase in the percentage of *Poldip2−/−* cells in the G1 and G2/M phases of the cell cycle, accompanied by a decrease in the percentage of S-phase cells. Increases in p53 S20 and Sirt1 were observed in passage 2 *Poldip2−/−* MEFs. In passage 4/5 MEFs, Cdk1 and CyclinA2 are downregulated in *Poldip2−/−* cells, and these changes are reversed by transfection with SV40 large T-antigen, suggesting that Poldip2 may target the E2F pathway. In contrast, p21^CIP1^ is increased in passage 4/5 *Poldip2−/−* MEFs and its expression is unaffected by SV40 transfection. Overall, these results reveal that Poldip2 is an essential protein in development, and underline its importance in cell viability and proliferation. Because it affects the cell cycle, Poldip2 is a potential novel target for treating proliferative conditions such as cancer, atherosclerosis and restenosis.

## Introduction

Polymerase delta interacting protein 2 (Poldip2, PDIP38, Mitogenin 1) is a ubiquitously expressed, 368 amino acid protein consisting of an N-terminal mitochondrial localization sequence and two main highly conserved functional domains: a ApaG/F box A domain and a hemimethylated DNA binding domain called YccV. It was originally identified as a binding partner of polymerase-δ and Proliferating Cell Nuclear Antigen (PCNA) [1]. Subsequent research has implicated Poldip2 in DNA replication and repair [2], [3], mitochondrial function and elongation [4], [5], and downstream signaling of a cell adhesion receptor [6], as well as cytoskeletal reorganization and regulation of reactive oxygen species production [7]. Our group reported that mice heterozygous for Poldip2 exhibit increased arterial stiffness and reduced aortic dilatation compared to wild type mice and exhibit increased collagen and disrupted elastic lamellae in arterial tissue [8], while homozygous deletion of Poldip2 results in perinatal lethality of unknown cause.

Several papers describe a possible role for Poldip2 in DNA replication/repair [1], [3], [9] or mitosis [2] that occurs during S-phase and M-phase, respectively. Poldip2 has been demonstrated to reduce polymerase δ activity in vitro [9]. Recent studies have implicated Poldip2 in the activity of translesional polymerases Polη, Rev1 and Rev7 [3]. Depletion of Poldip2 resulted in increased Polη foci in normal conditions and reduced cell survival after UV treatment. However, another study found that Poldip2 does not associate with PCNA or Polη foci after UV treatment of cells [10]. The authors instead propose that Poldip2 is involved in the processing of the DNA damage response protein MDM2, which may explain the reduced cell survival after UV treatment in Poldip2 depleted cells. However, there has been no study directly testing the role of Poldip2 in regulating the proteins involved in cell cycle progression, nor has its role in apoptosis, senescence and autophagy been investigated.

To better understand the functions of Poldip2, we used a mouse deficient in Poldip2. As previously described by our group, homozygous deletion of Poldip2 induces embryonic lethality [11]. Based on this observation, as well as the close relationship of Poldip2 to mechanisms regulating DNA synthesis and repair, we hypothesized that Poldip2 has multiple roles in cell division. We report here impaired growth in Poldip2 depleted cells, due in part to increased autophagy as well as altered expression of key cell cycle proteins such as Cyclin dependent kinase 1 (Cdk1), CyclinA2, Sirt1 and p21^CIP1^, suggesting that Poldip2 targets a common regulator such as E2F or p53.

## Methods

### Ethics statement

All animal protocols were approved by Institutional Animal Care and Use Committee of the Emory University School of Medicine.

### Animals

*Poldip2* gene trap mice in C57BL/6 background were produced by the Texas A&M Institute for Genomic Medicine (College Station, TX). A gene trap construct was inserted into the first intron of Poldip2 in mouse embryonic stem cells. The location of the gene trap was verified by polymerase chain reaction and sequencing. Mice were genotyped using a standard 3-primer PCR method. Phenotypic characterization of these mice has been published previously [8].

### Preparation of mouse embryonic fibroblasts (MEFs)

MEFs were prepared from E13.5 embryos. Briefly, female mice were euthanized by CO_2_ asphyxiation at day 13.5 post-conception. Using scissors, the abdomen was opened and the uterine horn was immediately removed intact and placed in PBS for dissection. Embryos were isolated with their yolk sacs intact. The yolk sac was removed and retained for genotyping. The head and internal organs of each embryo were removed and discarded. The dissected embryo was passed through an 18G needle to disperse the cells. The cells were plated on gelatin-coated 100-mm cell culture dishes in 15% FBS DMEM and passaged as described below.

### Cell culture

MEFs were grown in Dulbecco's Modified Eagle's Medium containing 15% fetal bovine serum (FBS). The cells were cultured using a 3T3 method; they were passed every 3 days and seeded at a density of 3×10^5^ cells per 20 cm^2^ dish. The cells were used for experiments between passages 2 and 7, at which point they became senescent.

### Growth curve/doubling curve

*Poldip2+/+*, *Poldip2+/−*, and *Poldip2−/−* MEFs at passages 0–6 were seeded at 10^4^ cells per 35 mm dish (Corning). Cells were trypsinized and counted every 24 h for 5 days using a Scepter 2.0 cell counter (Millipore). For the doubling curve, cells were counted at each passage and seeded at 3×10^5^ cells per 20 cm^2^. Population doublings after each passage were calculated as 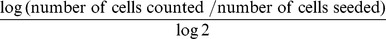. This value was added to that of previous passages to produce a cumulative doubling curve.

### Cell cycle analysis

MEFs were trypsinized 24 h after passage and fixed in 60% ethanol overnight. The cells were pelleted and washed with PBS. Cells were then resuspended in staining solution (1X PBS, 0.1% Triton-X, 0.2 mg/ml RNase A, 20 µg/ml propidium iodide (Sigma). Fluorescence signal was assessed using an LSRII (Becton, Dickinson) flow cytometer. Cell cycle analysis was performed using the Dean-Jett-Fox method in Flowjo (Treestar, Inc.).

### Apoptosis

MEFs were trypsinized 24 h after passage and fixed with 3% paraformaldehyde in PBS. Cells were stained with the apoptosis marker Annexin V, using the Annexin V:FITC Apoptosis Detection Kit I (Becton, Dickinson). Fluorescence signal was assessed using a LSRII flow cytometer (BD). Data was analyzed using Flowjo (Treestar, Inc.).

### MEF immortalization

*Poldip2+/+* or *Poldip2−/−* primary MEFs at passage 2 were seeded in 6-well plates (Corning). The cells were transfected with SV40 large T-antigen (Addgene plasmid 13970) using fugene HD (Promega). Cells were grown to confluence and transferred to 10-cm plates. Cells were then passaged at a ratio of 1∶10 for 9 additional passages upon reaching confluence.

### Western blot

Whole cell lysate was prepared from MEFs using radioimmunoprecipitation assay (RIPA) buffer (50 mM Tris, 150 mM NaCl, 1 mM EDTA, 0.1% SDS, 0.5% deoxycholate, 1% NP-40) with fresh protease and phosphatase inhibitors (PMSF, aprotinin, leupeptin, NaF, activated sodium orthovanadate). Protein concentrations were measured by Bradford assay, and protein was diluted into Laemmli buffer for separation by SDS-PAGE. Following separation, proteins were transferred to a nitrocellulose membrane and assessed by western blotting with primary antibodies against p21^CIP1^ (ab7960; Abcam), p27 (#25525; Cell Signaling), p53 (sc-99; Santa Cruz), p-p53 (S20) (sc-18078; Santa Cruz), β-actin (A5441; Sigma), Cdk1 (sc-54; Santa Cruz), Cdk2 (sc-163; Santa Cruz), Cdk4 (559693, BD), CyclinA2 (sc-751; Santa Cruz), CyclinB (#4138S; Cell Signaling), CyclinD1 (sc-718; Santa Cruz), CyclinE (sc-481; Santa Cruz), CyclinF (sc-953; Santa Cruz), E2F1 (sc-193; Santa Cruz), LC3b (#3868S; Cell Signaling), PCNA (ab2426; Abcam), Poldip2 goat antibody [7], Rb (#9313S; Cell Signaling), pRb(S780) (#9307S; Cell Signaling), pRb(S807/811) (#9308S; Cell Signaling), pRb(T821) (44-582G; Invitrogen), and Sirt1(#2028S; Cell Signaling). Blots were incubated with horseradish peroxidase (HRP)-conjugated secondary antibodies depending on the species of the primary antibody [anti-mouse (NA931; GE), anti-rabbit (170-6515; Bio-Rad), anti-goat (205-295-108; Jackson)], and assessed using enhanced chemiluminescence (ECL, GE). HRP-induced luminescence was detected with Amersham Hyperfilm ECL (GE). Detected bands were scanned and densitometry was performed using ImageJ.

### LC3I/II conversion assay for autophagy

MEFs were treated for 24 h with the protease inhibitors Pepstatin A (10 µg/ml) and E64d (10 µg/ml). Western blot was performed for LC3b as described above.

### Phosphoprotein purification and pRb detection

Phosphoproteins from MEFs in passages 2, 4, and 5 were purified using the Phosphoprotein Purification Kit (37101; Qiagen). Briefly, cells were rinsed with PBS and lysed in PhosphoProtein Lysis Buffer containing 10% CHAPS (Buffer 1) with added protease inhibitors and benzonase nuclease. Lysate was sonicated on ice and debris were pelleted by 10,000xg centrifugation for 30 minutes. Total protein concentration of the supernatant was quantified with a Bradford assay. The purification column was then equilibrated with Buffer 1. Protein was diluted to 0.1 mg/ml with Buffer 1 and added to the binding columns. The columns were washed with Buffer 1 and phosphoproteins were eluted with PhosphoProtein Elution Buffer containing 10% CHAPS. Eluate was concentrated with nanosep ultrafiltration columns and concentration was determined by Bradford. Protein was loaded on a 7.5% polyacrylamide gel and a western blot was performed as described above. Antibodies against Rb (10048-2-Ig; Proteintech) and pAkt (#9271S; Cell Signaling) were used for detection.

### RNA extraction and RT-qPCR

Total RNA was extracted with the RNeasy Plus kit (Qiagen). Reverse transcription was performed using Superscript II reverse transcriptase (Invitrogen) using random primers. cDNA was amplified with primers against Poldip2 (5′-TGCAGCTCCAGAAAAAGCAGAGAACC-3′, 3′-CTGACATAGTCCAAGCCTGGGATG-5′, Annealing temperature(Ta) – 60 °C), p21^CIP1^ (5′-GTCTGACTCCAGCCCCAAAC-3′, 3′-TGTGAGGACTCGGGACAATG-5′, Ta – 62 °C), p16^INK4a^ (5′-CCCAACGCCCCGAACT-3′, 3′-GCAGAAGAGCTGCTACGTGAA-5′, Ta – 62 °C), p19^ARF^(5′-TGAGGCTAGAGAGGATCTTGAGA-3′, 3′-TTGAGCAGAAGAGCTGCTACGT-5′, Ta – 65 °C) and Cdk1(5′-CTCTGGGCACTCCTAACAACGAA-3′, 3′-CAACACGATCTTCCCCTACGACCA-5′, Ta - 65 °C) in a buffer containing SYBR green by polymerase chain reaction using the LightCycler 1.1 (Roche) glass capillary real time thermocycler.

### Chromatin immunoprecipitation

MEFs in passages 2 and 5 were treated with 1% formaldehyde to crosslink proteins with DNA. ChIP was performed using the SimpleChIP kit (Cell Signaling). Briefly, cell were washed twice with PBS and harvested in PBS with PMSF. Cells were pelleted by centrifugation and lysed with buffer A containing DTT, PMSF, and protease inhibitors. After a 10-minute incubation on ice, the nuclei were pelleted and resuspended in buffer B with DTT. The resuspended nuclei were treated with 5 µl of micrococcal nuclease and incubated for 6 min at 37°C. The mixture was then centrifuged at 10,000xg and resuspended in 1 ml of ChIP buffer. The nuclei were lysed by sonication using a Microson Ultrasonic Cell Disruptor XL (Misonix, Inc) (2 cycles of 10, 1 second pulses at 4 watts). 10 µg of DNA-protein complexes were immunoprecipitated overnight at 4 °C using either p53 (sc-6243x; Santa Cruz), Histone H3 (#4620S; Cell Signaling) or IgG control (#2729P; Cell Signaling) antibodies. Immunocomplexes were precipitated by incubation for 2 h at 4 °C with ChIP-grade Protein G magnetic beads. Reversal of DNA-protein complex cross-linking was performed by incubation with ChIP elution buffer for 30 minutes at 65 °C. The samples were digested with proteinase K for 2 h at 65 °C. DNA was purified using the provided DNA binding columns. The DNA was used for qPCR amplification using the following primers surrounding the p53 binding site (−1971 to −1941) in the p21 promoter, (5′-CCAAGCCCTTCCCAGACTTCCA -3′ and 5′-GAGTTCTGACATCTGCTCTCCGAT-3′, Ta- 63°C). Each sample was normalized to 5% input DNA, quantified by qRT-PCR.

### Statistics

MEFs were prepared from unique embryos for each experiment. Data are presented as mean±SEM from a minimum of 3 independent experiments. Significance was determined using 2-way ANOVA followed by Bonferroni's post hoc test for multiple comparisons. A threshold of P<0.05 was considered significant.

## Results

### Poldip2 knockout results in reduced fetal weight and perinatal lethality

**Figure 1:**
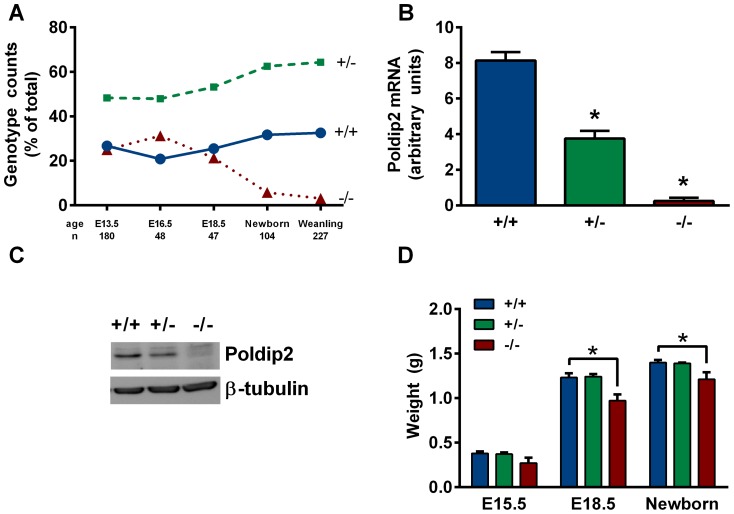
*Poldip2−/−* embryos exhibit perinatal lethality and reduced weight. (A) Progeny from heterozygote x heterozygote crosses were genotyped at different days post-conception and after birth. Mouse embryonic fibroblast Poldip2 (B) mRNA and (C) protein were measured to verify successful knockout. (D) Progeny at various stages of development were weighed and genotyped. Bars represent mean ± SEM of 3–4 independent mRNA experiments or 6–62 embryos or pups. * P<0.05 comparing *Poldip2+/+* with *Poldip2−/−*.

To clarify the role of Poldip2 *in vivo*, we used mice that have a gene trap construct inserted into the first intron of Poldip2, disrupting gene expression [11]. Expected Mendelian ratios would dictate that a cross of two heterozygotes should produce 25% wild type, 50% heterozygous and 25% homozygous animals; however, we observed 33% wild type, 64% heterozygous and 3% *Poldip2−/−* animals at birth (Figure 1A). To confirm the genotypes, mRNA (Figure 1B) and protein (Figure 1C) expression were measured in primary MEFs. As expected, Poldip2 mRNA and protein levels were about half that of wild type animals in heterozygotes, while *Poldip2−/−* animals had nearly undetectable levels of Poldip2.

We genotyped embryos at E13.5, E16.5, E18.5, and newborn stages to determine when *Poldip2−/−* embryos are lost. Surprisingly, *Poldip2−/−* embryos survive until birth (19.5 dpc) (Figure 1A), albeit at lower weight than wild type or heterozygous embryos (Figure 1D). There was no detectable weight difference between wild type and heterozygous embryos.

### Poldip2 knockdown causes reduced cell growth in MEFs

**Figure 2:**
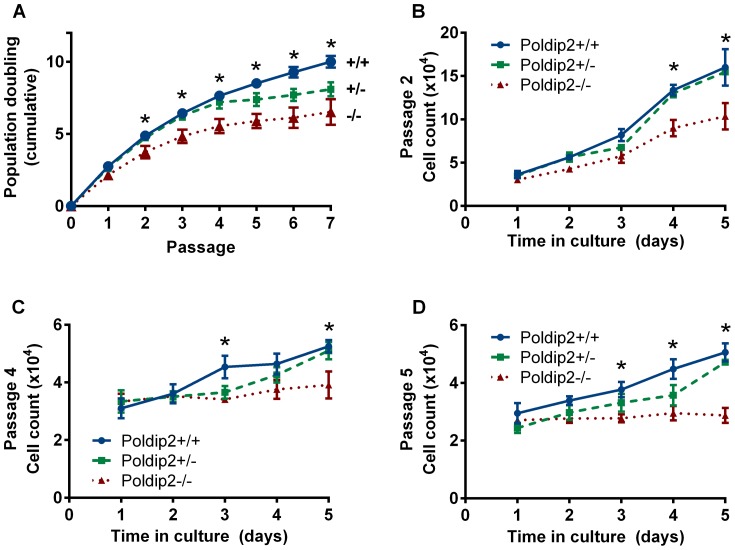
Reduced growth in Poldip2 null cells. Mouse embryonic fibroblasts were derived from *Poldip2+/+*, +/− and −/− E13.5 embryos. (A) Growth was assessed by counting cells at each passage and recorded as a cumulative population doubling. Additionally, growth was assessed by performing a growth curve at (B) passage 2, (C) passage 4, and (D) passage 5. Error bars represent mean ± SEM of 3–4 independent experiments. * P<0.05 comparing *Poldip2+/+* with *Poldip2−/−*.

Given the reduced weight of *Poldip2−/−* embryos, we sought to investigate the contribution of Poldip2 to cell growth. We prepared MEFs from E13.5 *Poldip2* +/+, +/−, and −/− embryos. Since the rate of growth of cells in culture can depend on inoculation density, we chose to maintain a common seeding density for the duration of the experiment. Cells were plated and passed according to the 3T3 method [12]. We compared the population doublings of cells of different genotypes (Figure 2A). The doubling of the wild type cells follows a predictable pattern of high growth in early passages, and slower growth at passage 5 or 6, which precedes the replicative senescence that naturally occurs in MEFs [13]. The *Poldip2*+/− cells show a pattern similar to the wild type, exhibiting slightly reduced growth that amounts to a difference of less than one doubling over seven passages. Strikingly, the *Poldip2−/−* MEFs have markedly reduced growth, which becomes obvious as early as passage 2. Over seven passages, the *Poldip2+/+* cells undergo three more doublings than the *Poldip2−/−* cells.

The growth of MEFs has been reported to be passage dependent, due to their propensity to senesce after 5–6 passages [14]. We chose to measure the difference in growth rate between *Poldip2+/+* and *Poldip2−/−* cells during several passages to investigate possible passage-dependent growth differences. *Poldip2−/−* cells grew significantly slower, which is most obvious in passage 2 (Figure 2B). By passages 4 (Figure 2C) and 5 (Figure 2D), cell growth has markedly slowed in all genetic groups. Wild type and heterozygous cells in later passages show reduced growth compared to early passage cells; however, the *Poldip2−/−* cells exhibit almost no growth in passage 4 or 5. We chose to concentrate on *Poldip2+/+* and *Poldip2−/−* cells for the remainder of the study. The early growth impairment in *Poldip2−/−* cells compared to *Poldip2+/+* led us to hypothesize that the lack of Poldip2 was leading to premature senescence, apoptosis and autophagy, or a block/delay in a cell cycle phase.

### Poldip2 knockout increases autophagy, but does not affect apoptosis or expression of senescence markers

**Figure 3:**
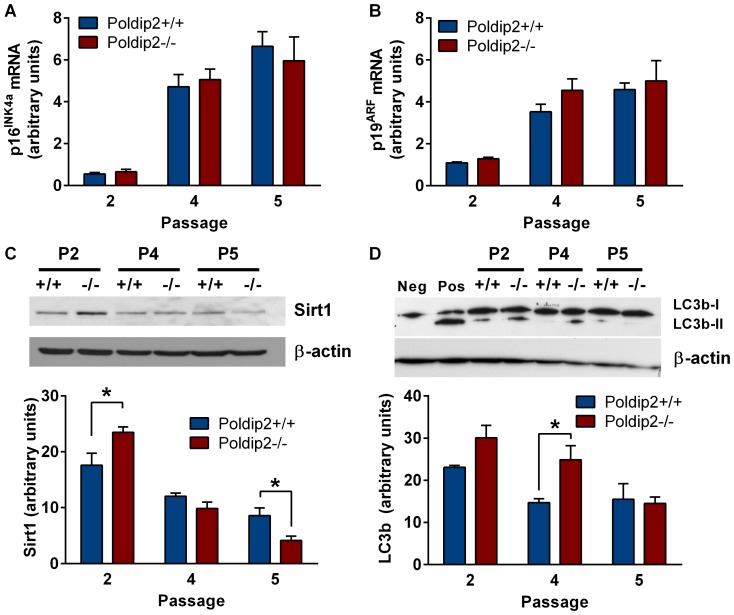
Poldip2 deletion increases Sirt1 expression and LC3b conversion but does not affect expression of senescence markers. (A) p16^INK4a^ and (B) p19^ARF^ mRNA expression was compared between *Poldip2+/+* (blue) and *Poldip2−/−* (red) cells by qRT-PCR and corrected for PPIA. (C) Sirt1 and (D) LC3b-1 and -II protein levels were assessed by western blot in Poldip2+/+ and Poldip2−/− MEFs at passages 2, 4 and 5 and normalized to β-actin. Error bars represent mean ± SEM of 3 independent experiments. * P<0.05 comparing *Poldip2+/+* with *Poldip2−/−*.

In order to determine whether *Poldip2*−/− cells enter senescence early, we measured the expression of senescence markers p16^INK4a^ (Figure 3A) and p19^ARF^ (Figure 3B) in passages 2–5. As expected, we observed an increase in the expression of p16^INK4a^ and p19^ARF^ as the passage number increased; however, there was no difference in the expression of senescence markers between genotypes. Because the gene trap construct in these mice includes a *lacZ* reporter, we were unable to use the β-galactosidase assay to confirm these findings. Nonetheless, it appears that lack of Poldip2 does not cause early senescence in MEFs. We additionally assessed the levels of Sirt1 in passages 2–5 and observed an increase in passage 2 (Figure 3C). There was no difference in Sirt1 in passage 4, but a significant decrease in passage 5.

Previous studies revealed interactions between Poldip2 and the p50 subunit of polymerase δ [1], polymerase η [3], and PCNA [1], which are involved in DNA replication and damage repair. A recent study also found Poldip2 interacts with polymerase γ, and reduction of Poldip2 by silencing can cause sensitivity to stress [15]. We therefore tested whether reduced growth in *Poldip2* knockout cells could be caused by increased apoptosis due to DNA damage. In passage 5 MEFs, there was a 9.1±1.3% basal level of Annexin V staining in wild type cells and 8.7±0.9% *Poldip2−/−* cells were Annexin V positive (n = 3, P = NS). The difference in growth between *Poldip2+/+* and *Poldip2−/−* cells thus does not appear to be due to increased apoptosis.

To determine whether enhanced autophagy contributes to the decrease in cell number observed in *Poldip2−/−* cells, we assessed the conversion of the autophagy marker LC3b-I to LC3b-II in p2-5 and observed increased LC3b-II in passage 4 (Figure 3D). This suggests that enhanced autophagy contributes to the observed reduction in growth at later passages.

### Poldip2 knockout arrests growth in G1 and G2/M

**Figure 4:**
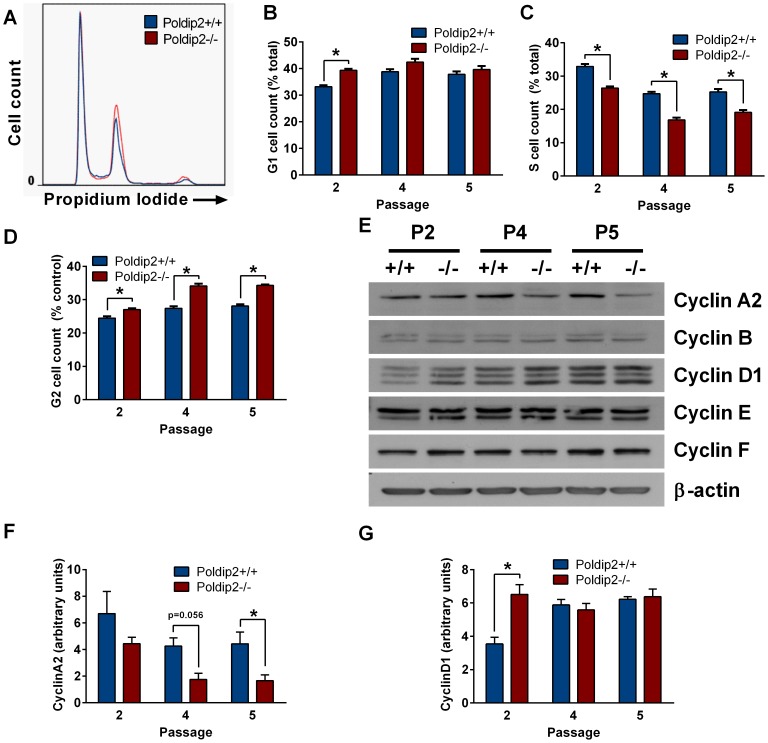
*Poldip2−/−* MEFs exhibit altered cell cycle characteristics. Asynchronous MEFs were collected at passage 2, 4 and 5 and stained with propidium iodide for FACS analysis of the cell cycle. (A) Passage 4 data is shown as an example histogram of DNA content as measured by flow cytometry and fit to the Dean-Jett-Fox model to calculate the percentage of cells in (B) G1, (C) S and (D) G2/M. (E) Key cell cycle protein expression was measured by immunoblotting. Protein levels were quantified by densitometry and corrected to β-actin expression for (F) CyclinA2 and (G) CyclinD1. All three bands were used in the quantification of CyclinD1. Error bars represent mean ± SEM of 3–4 independent experiments. * P<0.05 comparing *Poldip2+/+* with *Poldip2−/−*.

We next investigated the cell cycle distribution of *Poldip2+/+* and *Poldip2−/−* MEFs. We found that passage 2 *Poldip2−/−* MEFs are arrested or delayed in both G1 (Figure 4B) and G2/M (Figure 4D) phases of the cell cycle, resulting in a reduction of the number of cells in S-phase (Figure 4C). The genotype-related difference in the number of cells in G2/M, however, becomes greater in passages 4 and 5 compared to passage 2 cells.

To begin to investigate the mechanism underlying these changes in the cell cycle, we assessed protein levels of key cell cycle regulators (Figure 4E). Of the Cyclin proteins, only the expression of Cyclins A2 and D1 was significantly altered in Poldip2 null cells. CyclinA2 expression in passage 5 was significantly lower in *Poldip2−/−* cells than in *Poldip2+/+* cells (Figure 4F). Cyclin D1 was surprisingly higher in passage 2 *Poldip2−/−* cells than in *Poldip2+/+* cells, but was unchanged in later passages (Figure 4G). Taken together, these data demonstrate that there is differential regulation of the cell cycle associated with reductions of Cyclin A2 in *Poldip2−/−* cells.

### p53 phosphorylation and downstream targets are altered in Poldip2−/− MEFs

**Figure 5:**
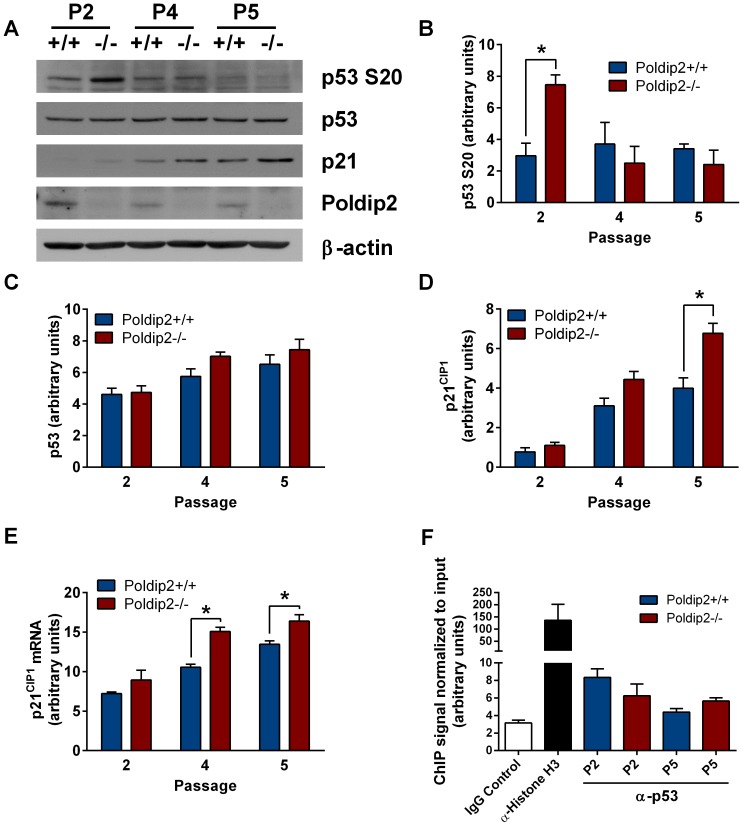
Poldip2 inhibits the p53 pathway. (A) Immunoblotting was performed using lysates from *Poldip2+/+* and *Poldip2−/−* MEFs in passages 2, 4 and 5. The blots were probed with antibodies against β-actin, Poldip2, p53, phospho-p53(S20), and p21^CIP1^. Densitometry was performed and corrected to β-actin (B, C, E). (D) p21^CIP1^ mRNA levels were assessed by RT-qPCR and corrected for the housekeeping gene PPIA. (F) ChIP was performed on *Poldip2+/+* (blue) and *Poldip2−/−* (red) MEFs using p53 antibody and p21^CIP1^ promoter primers. Poldip2+/+ cells were used for the IgG negative and Histone H3 antibody positive controls. All samples were normalized to input DNA. Error bars represent mean ± SEM of 3–4 independent experiments. * P<0.05 comparing *Poldip2+/+* with *Poldip2−/−*.

Because previous studies indicate that the p53 pathway can cause a delay in G1 and G2/M [16], we measured expression of p53 and p21^CIP1^, a p53 transcription target, by western blot. Total p53 was unchanged (Figure 5A, C), but phospho-p53 (S20), an indicator of p53 activity, was markedly increased in *Poldip2−/−* cells compared to wild type cells at passage 2, but not at later passages (Figure 5B). As noted above, the deacetylase Sirt1, which is also activated by p53, was increased in passage 2 as well (Figure 3C). In contrast, p21^CIP1^, a cell cycle inhibitor positively regulated by p53, is significantly increased only at later passages at both the protein (Figure 5E) and mRNA levels (Figure 5D). In order to determine whether p53 binding to the p21^CIP1^ promoter is altered with Poldip2 depletion, we performed a ChIP assay in passages 2 and 5 (Figure 5F). There was no observed change in p53 binding at either passage. Together with the observation that Sirt1, which in addition to being activated by p53 acts as a negative regulator of p53 activity, these data can be interpreted as activated p53 having a delayed effect upon p21^CIP1^ transcription, or that another transcription factor or epigenenetic change is responsible for the observed changes in p21^CIP1^. Nevertheless, p53/Sirt1 may play a role in the growth response at passage 2.

### E2F target genes exhibit reduced expression in Poldip2−/− cells

**Figure 6:**
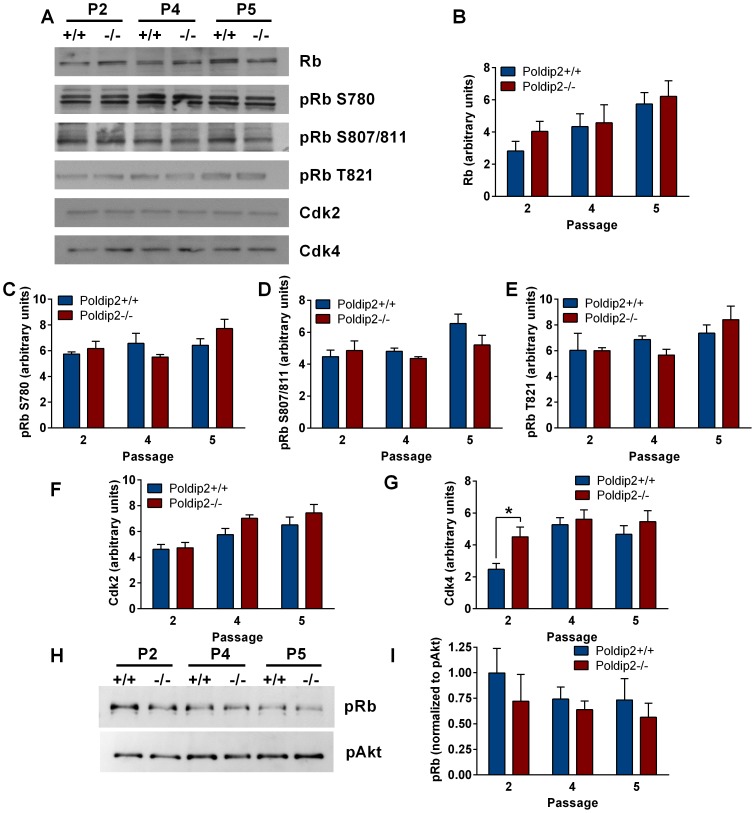
Poldip2 deletion does not affect Rb expression/phosphorylation. (A) Immunoblotting was performed using lysates from *Poldip2+/+* and *Poldip2−/−* MEFs in passages 2, 4 and 5. The blots were probed with antibodies against Rb, pRb S780, pRb S807/811, pRb T821, Cdk2, and Cdk4. Densitometry was performed and corrected to β-actin run in parallel (shown in Figure 7A) (B–G). (H) Phosphorylated proteins from Poldip2+/+ and Poldip2−/− cells in passages 2, 4 and 5 were purified with a phosphoprotein binding column. Phosphorylated Rb was measured by immunoblot and normalized to pAkt levels (I). Error bars represent mean ± SEM of 3–4 independent experiments. * P<0.05 comparing *Poldip2+/+* with *Poldip2−/−*.

The data in Figure 4 indicate a significant decrease in CyclinA2 levels in p4 and p5 after *Poldip2* deletion. CyclinA2 expression is positively regulated in part through the E2F transcriptional activators and their binding partner, Rb, which prevents E2F-dependent transcriptional activity. Total protein levels of Rb, the main regulator of E2F, were unchanged (Figure 6A, B). However, Rb binding with E2F is regulated by its phosphorylation state. Two of the kinases responsible for phosphorylating Rb in G1 are Cdk2 and Cdk4. Whereas Cdk2 was unchanged (Figure 6F), Cdk4 was significantly increased in p2 *Poldip2−/−* cells compared to control cells (Figure 6G). Interestingly, the cyclin binding partner of Cdk4, CyclinD1 was similarly increased in p2 *Poldip2−/−* cells (Figure 4E). Unexpectedly, we did not see increased phosphorylation of Rb sites that would indicate elevated Cdk2 (T821) and Cdk4 (S780, S807/811) activity [17]. In fact, none of the sites measured, pRb S780 (Figure 6C), pRb S807/811 (Figure 6D), or pRb T821 (Figure 6E) showed a significant difference in phosphorylation between *Poldip2+/+* and *Poldip2−/−* cells. To confirm these results, we performed a phosphoprotein purification to identify whether untested Rb phosphorylation sites were altered by loss of Poldip2. There was no change in Rb phosphorylation levels at any passage (Figure 6H).

**Figure 7:**
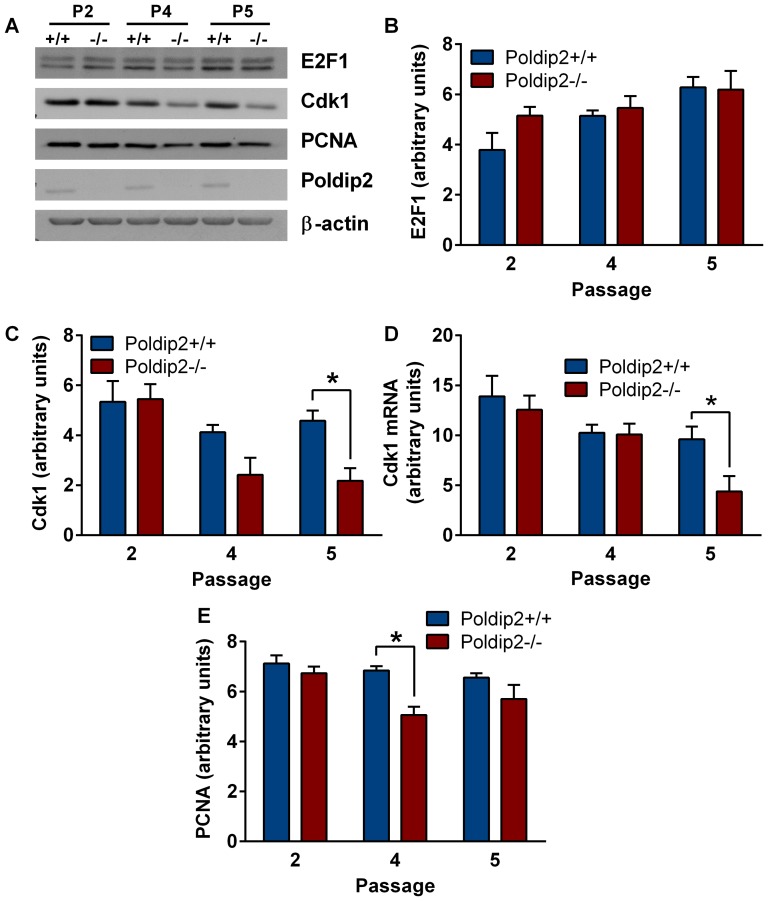
Poldip2 activates the E2F1 pathway. (A) Protein levels of E2F1 target genes were measured by immunoblot. Lysates from *Poldip2+/+* and *Poldip2−/−* cells in passages 2, 4 and 5 were probed with antibodies against β-actin, Poldip2, E2F1, Cdk1 and PCNA. Densitometry was performed and corrected to β-actin (B–D). (E) Cdk1 mRNA levels were measured by RT-qPCR and corrected with PPIA. Error bars represent mean ± SEM of 3–4 independent experiments. * P<0.05 comparing *Poldip2+/+* with *Poldip2−/−*.

Total E2F1 levels were also unchanged (Figure 7B). However, another E2F target, Cdk1 (Figure 7C), was downregulated in p5 in *Poldip2−/−* compared to wild type cells. mRNA of Cdk1 was measured to verify that the change in protein was a result of changes in mRNA expression (Figure 7E), and indeed Cdk1 mRNA was reduced at passage 5 in *Poldip2−/−* cells compared to controls. Moreover, the E2F target protein and Poldip2 binding partner PCNA was significantly reduced in p4 (Figure 7D). These data suggest that Poldip2 positively regulates canonical E2F targets, including Cdk1, in a direct or indirect fashion.

### SV40 immortalization restores Poldip2 growth to wild type levels

**Figure 8:**
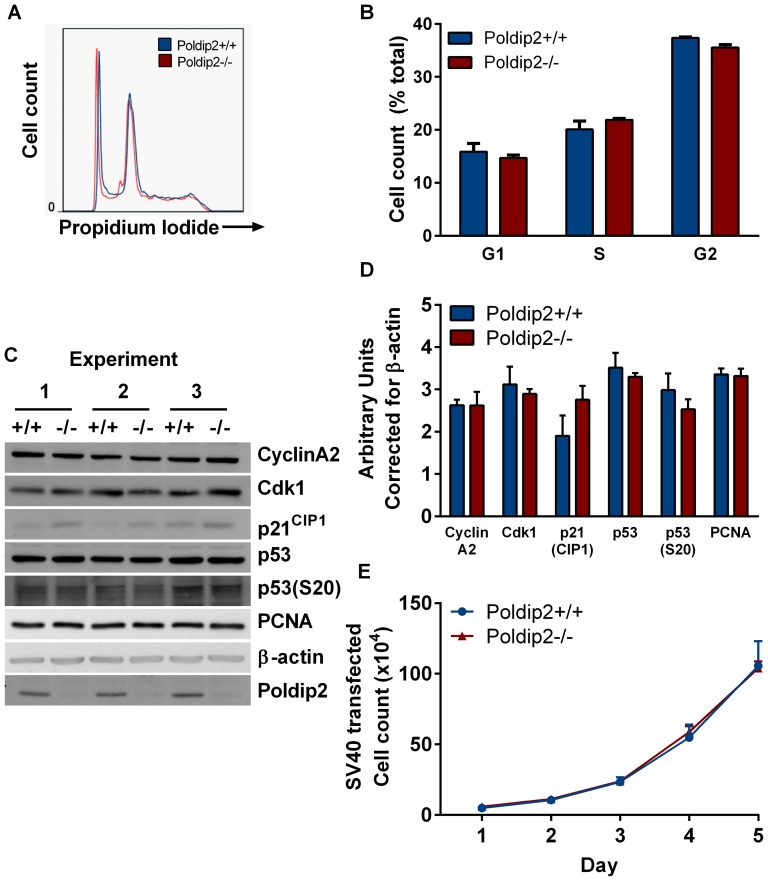
SV40 immortalization of *Poldip2−/−* MEFs restores growth and cell cycle distribution. *Poldip2* +/+ and −/− MEFs were immortalized with SV40 large T-antigen. (A, B) Cell cycle analysis was performed as in Figure 4 using flow cytometry. (C) Expression of the indicated proteins was assessed by western blot in 3 independent batches of immortalized cells. (D) Densitometry was performed and corrected to β-actin. (E) A growth curve was performed to compare *Poldip2+/+* (blue line) to *Poldip2−/−* (red line) MEFs. Error bars represent mean ± SEM of 3 experiments.

Due to the effects on p53 and Rb/E2F downstream targets, we hypothesized that inactivating Rb and p53 by expressing SV40 large T-antigen in *Poldip2−/−* cells would rescue the deficiency in proliferation. SV40 LTA has been previously demonstrated to bind and sequester Rb, which allows E2F to bind DNA in its active state [18]. Wild type and *Poldip2−/−* MEFs were transfected with SV40 large T-antigen at passage 2. Cell cycle distribution analysis of the cells by flow cytometry (Figure 8A) showed that expression of SV40 large T-antigen in *Poldip2−/−* cells restored cell cycle distribution to the wild type pattern (Figure 8B). Immortalization with SV40 also prevented the impairment of growth induced by loss of Poldip2 (Figure 8E). Protein levels of the E2F target genes CyclinA2, Cdk1 and PCNA were restored to wild type levels (Figure 8C and 8D). Phosphorylation of p53 S20 was not readily detectable, but p21^Cip1^ levels showed a trend towards being elevated in SV40 immortalized *Poldip2−/−* MEFs, similar to untransformed *Poldip2−/−* MEFs (Figure 8D and 5D). These results indicate that SV40 immortalization is sufficient to overcome the effect of Poldip2 loss on E2F-dependent cell cycle regulators and proliferation.

## Discussion

**Figure 9:**
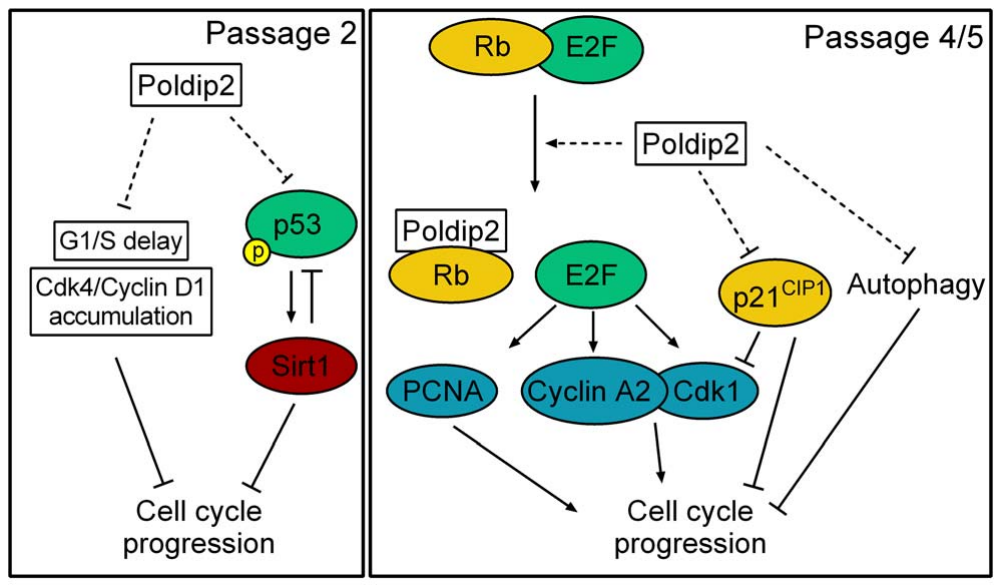
Proposed mechanism by which Poldip2 promotes cell cycle progression. Left: Passage 2. Poldip2 promotes progression though G1/S by preventing the accumulation of Cdk4/Cyclin D1 that occurs in *Poldip2−/−* cells. Poldip2 also limits the activation of p53 by phosphorylation at S20, which results in reduced Sirt1 expression. Reduced Sirt1 negatively regulates p53 by de-acetylation and reduces cell cycle progression. Right: Passage 4/5. Poldip2 activates the transcription of E2F target genes such as Cyclin A, Cdk1 and PCNA. These act to promote cell cycle progression. Poldip2 also reduces expression of the cell cycle inhibitor p21^CIP1^. Reduced p21^CIP1^ promotes cell cycle progression by relieving inhibition of the activity of Cyclin/Cdk complexes. We propose that Poldip2, like SV40 immortalization, inhibits p53 activity and sequesters Rb away from E2F, promoting cell cycle progression. Finally, Poldip2 inhibits autophagy, which results in increased growth.

In this study, we uncovered a novel role of Poldip2 in cell cycle regulation (Figure 9). We demonstrated that mice lacking Poldip2 are smaller during embryonic development and suffer perinatal lethality. The loss of Poldip2 reduces growth of MEFs, increases autophagy and changes the cell cycle distribution of asynchronous cells. Poldip2 depletion increases p53 S20 phosphorylation and Sirt1 protein expression in passage 2 and increases expression of p21^CIP1^ in passages 4 and 5. Additionally, E2F/Rb-dependent gene expression is repressed in *Poldip2*−/− cells as evidenced by the loss of PCNA, CyclinA2 and Cdk1 in passages 4 and 5. Finally, we showed that the cell cycle delays and expression of cell cycle regulators resulting from the loss of Poldip2 can be rescued by inhibiting p53 and Rb with SV40 LTA expression. This is the first time that Poldip2 has been reported to be implicated in cell cycle checkpoint regulation.

These results at first seem to contradict flow cytometry analysis performed in a previous study of Poldip2, which found no difference in the cell cycle after treatment with siRNA against Poldip2 [3]. However, that study was performed in SV40-transformed human fibroblasts. Our data clearly show that SV40 transformation eliminates cell cycle alterations that are readily apparent in primary MEFs. In fact, many experiments in earlier publications describing Poldip2 function and localization were performed in immortal or cancer cell lines such as HEK293 [1], [9], HeLa [1], [4], [6], [9], and C2C12 [5]. These results may need to be reexamined in primary cells to verify that localization and function of Poldip2 were not altered by the immortalization or transformation. Interestingly, one of the few Poldip2 studies performed in primary cells (rat brain endothelium) finds alterations in mitosis related to chromosome segregation defects [2]. In that study, Poldip2 antibody injections and siRNA were demonstrated to cause disorganized spindles, disrupted chromosomal segregation and multinucleated cells. Consistent with our results, the authors suggest that Poldip2 likely has multiple interacting partners and might be involved in the control of a cell cycle checkpoint, which could explain the observed defect; however they did not assess cells outside of mitosis. The present work provides direct evidence that Poldip2 does in fact regulate cell cycle progression.

In this study, we focused on key cell cycle regulatory pathways in early (p2) or late (p4/p5) passage MEFs. Growth curves indicate that *Poldip2−/−* MEFs grow slower than *Poldip2+/+* MEFs during passages 2–5. We also observed an accumulation of *Poldip2−/−* cells in G1 and increased Cdk4/CyclinD1 protein expression in passage 2. Cdk4 and CyclinD1 typically accumulate at the G1/S transition to promote the initiation of DNA synthesis. In this case, their increased expression could be due to the increased number of *Poldip2−/−* cells in G1 in passage 2. Given recent publications describing Poldip2's potential involvement in DNA damage repair, we investigated the tumor suppressor p53, which is often activated in cases of DNA damage and other stress. Although we do not observe a change in overall p53 expression, it has been established that expression alone is not the determinant of transcription activity [19]. Posttranslational modifications such as phosphorylation, ubiquitination and acetylation influence the expression and activity of p53 [20]. In passage 2 *Poldip2−/−* MEFs, we observed an increase in p53 phosphorylation at serine 20 and increased protein expression of Sirt1, a p53 transcriptional target. Sirt1 has also been shown to act as a negative regulator of p53 activity by deacetylating it [21]. Although it is controversial whether or not Sirt1 is an oncogene, a study of a Sirt1 knockout mouse supports a role as a tumor suppressor [22]. Additionally, resveratrol, an activator or Sirt1, has been demonstrated to be effective as a therapy for some cancers [23]. Elevated Sirt1 expression in passage 2 *Poldip2−/−* MEFs could explain why increased p53 S20 phosphorylation does not increase p53 binding or p21^CIP1^ expression in passage 2. Although we found increased p21^CIP1^ expression in passages 4 and 5, we did not observe increased binding of p53 to the p21^CIP1^ promoter in p2 or p5 by ChIP assay. The observed increase in p21^CIP1^ in *Poldip2−/−* cells is likely mediated by another transcription factor such as C/EBP, Sp1, STAT, Smad, BRCA1 or AP2 [24]. One possible scenario is that Poldip2 induces an increase in autophagy, which has been demonstrated to cause an increase in p21^CIP1^ via p38 MAPK activation of STAT [25]. Indeed, in passage 4 we observed an increase in LC3b-II, a marker of autophagy, when the elevation in p21^CIP1^ was first identified.

The influence of Poldip2 on CyclinA2 and Cdk1 was not observed until passage 4. There is a precedent for Cdk1 reduction only in later passages of knockout MEFs: in a Cdk2/Cdk4 double knockout study, Berthet et al. [26] observed a similar late decrease in Cdk1 protein and Cyclin A2. This was found to be due to a hypophosphorylation of Rb. Hypophosphorylation of Rb results in its increased binding to E2F, reducing the transcription of cell cycle regulators, including Cdk1 and CyclinA. We did not observe a change in Rb phosphorylation in *Poldip2−/−* MEFs. However, Rb binding to E2Fs could be altered in other ways. If Poldip2 directly binds Rb, it could prevent Rb mediated inhibition of E2Fs independent of Rb phosphorylation, acting as a redundant mechanism to Rb phosphorylation. Both HPV E7 and SV40 large T-antigen sequester Rb in this manner, resulting in immortalization of many cell types [18]. Another possibility is that Poldip2 binds directly to E2Fs, preventing Rb from binding even in a hypophosphorylated state. Poldip2 could also aid E2Fs in binding DNA; indeed, one of Poldip2's domains (YccV) is a DNA binding domain in bacteria [27]. At this time the mechanism of Poldip2's effect on E2F activity is unclear; further study is necessary to elucidate the effect of Poldip2 on E2F target gene expression.

The E2F/Rb transcriptional pathway has been of much interest in cancer biology, due to its complex regulation of the cell cycle and apoptosis. One of the desirable methods of targeting cancer has been to overcome tumor cell resistance to senescence and apoptosis, while leaving normal cells untouched. The Cyclin dependent kinases have been the targets of drug and genetic therapies because Cdks play such a key role in cell cycle regulation [28]. The role of Poldip2 in tumor formation is not well known yet, although a study of the sense-antisense gene pair of TNFAIP1/POLDIP2 found poor prognosis in breast cancer patients with upregulated Poldip2 expression [29]. A second key area where antiproliferative research can make an impact on is the prevention of post-angioplasty restenosis. A recent trial found that bare stents have a 21% restenosis rate within 24 months [30]. Antiproliferative drugs in a drug eluting stent could lower recurrence rates by limiting vascular smooth muscle cell growth after angioplasty. We recently found that depletion of Poldip2 in vascular smooth muscle cells also results in impaired proliferation (unpublished observations), supporting a potential role of Poldip2 in restenosis.

Our current understanding of Poldip2 is far from complete. Future studies are necessary to uncover exactly how Poldip2 influences the cell cycle and E2F target proteins. However, this study and others highlight the importance of Poldip2 in growth, including the cell cycle and DNA duplication/repair [1]–[3], [9]. Perinatal lethality in the absence of Poldip2, as well as the reduced growth in *Poldip2−/−* primary cells, indicates that Poldip2 is essential for normal cell growth and proliferation. A further understanding of Poldip2 signaling may uncover novel targets for antiproliferative drugs, and provide a better understanding of the mechanism of current therapies.
